# Endoscopy of Low BMI Patients Compared to Normal BMI Patients: A Study From the Aseer Region, Saudi Arabia

**DOI:** 10.7759/cureus.61276

**Published:** 2024-05-28

**Authors:** Yahia Al-Hagawi, Nasser I Alqahtani, Saeed Nasser Alsharif, Rafaat Chakik, Dawlah Hadi Asiri, Salihah Y Al mani, Azizah Badawi, Haneen Ahmad Al-assiri, Hana Saeed Al Malih, Hend Alamri, Amjad Saad AlAli, Aisha A Ali Alqhtani, Asiah A Al-BinAbdullah, Mohamed H Elgazzar

**Affiliations:** 1 Gastroenterology, Armed Forces Hospital Southern Region (AFHSR), Khamis Mushait, SAU; 2 Internal Medicine, Armed Forces Hospital Southern Region (AFHSR), Khamis Mushait, SAU; 3 Medicine, Ahad Rufaida Hospital, Khamis Mushait, SAU; 4 Medicine, Billasmar Hospital, Khamis Mushait, SAU; 5 Internal Medicine, Hepatology, and Gastroenterology, Faculty of Medicine, Mansoura University, Mansoura, EGY

**Keywords:** risk variables, severe adverse events, post-endoscopy complications, low bmi, gastrointestinal endoscopies

## Abstract

Gastrointestinal (GI) endoscopies are essential for detecting and treating various digestive tract problems. While typically safe, these treatments can entail the risk of severe adverse events (SAEs), especially in individuals with a low body mass index (BMI). The current study aimed to evaluate whether post-endoscopy SAEs are more common in patients with low BMI and find risk factors for serious adverse outcomes in Saudi Arabian patients from Khamis Mushait, Aseer region, Saudi Arabia. The data of 398 adult patients with abdominal endoscopies between April and November 2023 were analyzed. Patients were divided into two groups: low BMI (BMI ≤ 18.5) and control (18.5 ≤ BMI ≤ 30). They were matched for age, gender, comorbidities, endoscopy type, and other pertinent characteristics. Low-BMI patients (Group I, n = 108) were substantially younger and had lower levels of albumin and total protein than the control group (Group II, n = 209). Comorbidities varied between groups, with diabetes mellitus more prevalent in Group II and inflammatory bowel disease (IBD) more commonplace in Group I. Treatment options also differed, with Group I receiving more biological treatments, steroids, and feeding tubes. Endoscopic procedures and indications were comparable among groups, with no significant variations in post-endoscopy complications. The endoscopy results varied from gastritis to colon malignancy, with no SAEs recorded in either group. Unlike earlier findings, this study found no higher incidence of SAEs in low-BMI individuals having abdominal endoscopy. This might be because of the restricted guidelines of different medical authorities, including clear informed consent that illustrates any risks, benefits, alternatives, sedation plan, and potential diagnostic or therapeutic interventions. Also, professional endoscopists and consultants who ensure adequate visualization of the GI mucosa, using mucosal cleansing and insufflation as necessary, should avoid any risk of abdominal hemorrhage. These findings highlight the significance of personalized risk assessment and pre-procedural optimization, including nutritional assistance, in this patient population. More prospective research with larger sample sizes is needed to validate these findings and create targeted techniques for improving outcomes in individuals with a low BMI having endoscopic operations.

## Introduction

Gastrointestinal (GI) endoscopies are critical diagnostic procedures that examine the inner lining of the digestive tract. This minimally invasive treatment includes inserting a flexible tube with a camera and light source into the mouth or rectum, letting doctors see and assess the esophagus, stomach, small intestine, and colon [1]. Endoscopies are used to diagnose various GI disorders, including ulcers, polyps, inflammation, bleeding, and cancer. Furthermore, they allow therapeutic procedures like polyp excision, hemorrhage management, and stent implantation [2]. In the general population, they are regarded as reasonably safe, with the incidence of adverse events (AE) increasing with procedure complexity. With advancements in technology and techniques, GI endoscopies have become safer, more efficient, and increasingly integral to providing precise diagnoses and treatments for patients with GI disorders [3,4].

Severe adverse events (SAEs) after colonoscopy include a variety of possible problems. These include GI perforation, which may result from mechanical forces against the bowel wall, barotrauma, or a direct result of therapeutic procedures. Early symptoms include persistent abdominal pain and abdominal distention [5]. Another prominent consequence is GI bleeding, which occurs when polyps are removed during a colonoscopy or when the colon lining is traumatized, necessitating bleeding management procedures [6]. Although rare, sedation-related cardiopulmonary problems such as respiratory depression or cardiovascular events might occur, emphasizing the significance of meticulous monitoring throughout the treatment. Furthermore, post-polypectomy syndrome, which is characterized by stomach discomfort, fever, and leukocytosis, can develop following polyp removal, but rarely. Infection, albeit uncommon, is a possible concern following colonoscopy, especially if correct sterile methods are not used [7]. Patients should be educated about these potential consequences and closely monitored by healthcare professionals to reduce risks and guarantee prompt treatment if SAEs occur.

Indeed, the risk factors for SAEs after colonoscopy can be complex, involving both patient- and procedure-related characteristics. Patient-related risk factors include advanced age since elderly people may have weaker colonic walls and are more susceptible to problems [8,9]. The female gender has also been linked to a slightly greater incidence of SAEs, either due to anatomical variations or hormonal reasons [10]. Comorbidities such as cardiovascular disease, diabetes, or chronic renal disease might increase the risk of problems during and after the treatment. Medications, including anticoagulants and antiplatelet medications, can increase the risk of bleeding during polypectomy [9].

Procedure-related risk factors include the reason for colonoscopy, with emergency procedures posing a greater risk than elective ones. Prolonged procedure times, generally owing to complicated anatomy or difficult polyp removal, might increase the risk of adverse outcomes [11]. The performance of polypectomy itself is a substantial risk factor, especially if big or numerous polyps are removed, as this increases the likelihood of bleeding or perforation. Furthermore, the size and position of polyps are important, with bigger or sessile polyps in difficult places offering a higher risk [12].

Underweight individuals, defined as having a body mass index (BMI) of less than 18.5, are a vulnerable demographic population [13]. When regular endoscopic treatments are required, these people frequently confront specific obstacles and considerations due to their decreased body weight and possible underlying health issues. Patients with LBMI are predisposed to sharp angulations during the endoscopic encounter, thus increasing the likelihood of trauma. The patients are also associated with difficult colonic intubation, longer colon, and more chances of looping, thus increasing the overall contact time of the endoscope with the mucosal surface and increasing the shear stress within the lumen [14,15].

Sedation and anesthesia during endoscopic operations are particularly concerning for underweight patients. Their decreased body weight may necessitate cautious medication dose adjustments to avoid over-sedation or unpleasant responses. Furthermore, underweight people may be more vulnerable to aspiration during sedation due to a lack of respiratory reserve and muscular mass [7,16].

Healthcare practitioners performing endoscopic operations on underweight people should be aware of these risks and take necessary precautions to guarantee their patients' safety and well-being. This may involve close monitoring during and after the treatment, customized sedation regimens, and post-procedure nutritional assistance, if necessary. Furthermore, a thorough pre-procedure examination and optimization of nutritional status and general health may assist in reducing risks and enhancing results for underweight patients having endoscopic operations. So, the current study aimed to evaluate whether post-endoscopy SAEs are more common in patients with low BMI and find risk factors for serious adverse outcomes in patients from Saudi Arabia.

## Materials and methods

Study type and design

A single-center study was conducted on patients visiting the internal medicine department from April 2023 to November 2023. Patients had endoscopic procedures for different medical purposes. All patients were adults (age ≥ 18 years at the time of endoscopic interventions) and had an endoscopic intervention. Patients were divided into two groups: Group I, which had a low BMI (BMI ≤ 18.5%), and Group II, which is the control group (18.5 ≤ BMI ≤ 30%).

Both groups were matched with regard to age, gender, comorbid disease, type of endoscopy, inflammatory bowel disease (IBD) diagnosis, a diagnosis of malignant disease of any kind, use of anticoagulation drugs (for a week pre-procedure), and any prior abdominal or pelvic surgery.

The sample size was calculated as previously described by Kadam and Bhalerao [17]. So, 398 patients were recruited from one medical center in Saudi Arabia.

Inclusion criteria

Males and females aged 18 years or older at the time of endoscopic interventions and all patients with BMIs lower than 30% were included in the study. These patients were then divided into a low BMI (LBMI) group, defined as BMI ≤ 18.5%, and a sample comparator group (normal-overweight BMI range, NBMI), which included patients with BMIs higher than 18.5% and lower than or equal to 30%. These patients underwent endoscopic intervention from April 2023 to November 2023.

Exclusion criteria

Males and females aged less than 18 years at the time of endoscopic interventions and patients with a BMI value > 30% were excluded from the study.

Data collection

Data were collected from the medical records of the participating center. It was defined as the information collected and generated by a healthcare provider to provide healthcare for the personal benefit of the patient, where the information within medical records has clinical validity and utility.

Data were collected and extracted from original data sources anonymously on a patient-level basis and then combined to conduct statistical analysis. These data included age, gender, weight, height, BMI, comorbidities, treatments, laboratory results pre-endoscopy, endoscopy interventions, endoscopy indications, endoscopy diagnosis, and post-endoscopy complications.

Statistical analysis

The data gathered underwent organization, tabulation, and statistical analysis using SPSS software version 22 (IBM Corp., Armonk, NY). Mean and standard deviation (SD) calculations were conducted for quantitative data. Whenever relevant, one-way ANOVA was employed to compare the means of categories of two or more variables to determine any statistical evidence between the two groups. Qualitative data were summarized through calculations of frequency and percentage distributions. A significance threshold of p ≤ 0.05 was applied to interpret the results of significance tests.

## Results

In the present study, the medical records of 398 patients who underwent abdominal endoscopy were investigated. The demographic and clinical characteristics are listed in Table 1. Among them, 108 patients had a lower BMI (LBMI), while 209 had normal BMI values (control). Patients in the LBMI group had significantly lower ages (39.1 ± 19.7 years) than those in the control group (50.1 ± 16.6 years) (p < 0.001). Statistically, none of the collected data followed a normal distribution regarding the BMI categories. The statistical analysis revealed significant variance between both groups regarding gender (p = 0.013).

**Table 1:** Demographic and clinical characteristics of the studied patients ^#^ One-Way ANOVA (POSTHOC = LSD ALPHA [0.05]). * Significant at p < 0.05. BMI: Body mass index; Hb: Hemoglobin; WBC: White blood cells; Plt: Platelets; sCr: Serum creatinine; Alb: Albumin; CKD: Chronic kidney disease; CVA: Cerebrovascular accident (stroke); CVD: Cerebrovascular disease; COPD: Chronic obstructive pulmonary disease; IBD: Inflammatory bowel disease; OR: Odds ratio; CI: Confidence of intervals; PPI: Proton pump inhibitors; LSD: Least significant difference.

Variables		LBMI group (N = 108)	Control group (N = 290)	P-value^#^	OR	95% CI	
						Low	Up
Age (years)		39.1 ± 19.7	50.1 ± 16.6	<0.001*			
Weight (kg)		47.4 ± 5.5	64.3 ± 9	<0.001*			
Height (cm)		163.1 ± 6	162.8 ± 7.5	0.688			
BMI (%)		17.7 ± 0.9	24.2 ± 2.5	<0.001*			
Gender	Male	49 (45%)	172 (59.3%)	0.013*	0.57	0.37	0.89
	Female	59 (54%)	118 (40.7%)				
Laboratory results	Hb (g/dL)	12.2 ± 2	12.6 ± 2.1	0.069			
	WBC (×10^9^/L)	6.6 ± 1.9	6.6 ± 1.9	0.961			
	Plt (×10^9^/L)	291.9 ± 90.9	283.5 ± 91.1	0.414			
	sCr (µmol/L)	85.5 ± 74.5	91.9 ± 71.3	0.435			
	Alb (g/L)	35.8 ± 6.4	38.1 ± 4.7	<0.001*			
	Total protein (g/L)	70.4 ± 6.9	73.3 ± 5.1	<0.001*			
Comorbidities	None	48 (44.4%)	132 (45.7%)	0.827	0.95	0.61	1.48
	CKD	14 (13%)	41 (14.1%)	0.763	1.1	0.58	2.12
	CVA/Dementia	10 (9.3%)	11 (3.8%)	0.03*	0.39	0.16	0.94
	CVD	6 (5.6%)	27 (9.3%)	0.228	1.75	0.7	4.35
	COPD	0 (0%)	1 (0.3%)	0.542	1	1	1.01
	Connective tissue diseases	6 (5.6%)	13 (4.5%)	0.656	0.8	0.3	2.16
	Liver disease	6 (5.6%)	19 (6.6%)	0.717	1.19	0.46	3.07
	Diabetes	28 (25.9%)	121 (41.7%)	0.004*	2.05	1.25	3.34
	Malignancy	3 (2.8%)	11 (3.8%)	0.626	1.38	0.38	5.04
	IBD	17 (15.7%)	15 (5.2%)	0.001*	0.29	0.14	0.61
Previous abdominal pelvic surgery		18 (16.7%)	62 (21.4%)	0.697			
Treatment	None	48 (44.4%)	135 (46.7%)	0.687	0.91	0.89	1.42
	Chemotherapy	3 (2.8%)	10 (3.4%)	0.739	1.25	0.34	4.63
	Biological	17 (15.7%)	15 (5.2%)	0.001*	0.29	0.14	0.61
	PPI	39 (36.1%)	126 (43.3%)	0.187	1.36	0.86	2.15
	Antibiotics	8 (7.4%)	11 (3.8%)	0.133	0.49	0.19	1.26
	Steroids	18 (16.7%)	20 (6.9%)	0.003*	0.37	0.19	0.73
	Anticoagulants	7 (6.5%)	22 (7.6%)	0.707	1.18	0.49	2.86
	Feeding tube	5 (4.6%)	2 (0.7%)	0.008*	0.14	0.03	0.75

The history of clinical records before the endoscopy procedure comprised three categories: comorbidities, laboratory results, and prescribed treatments. LBMI patients had significantly lower levels of albumin (35.8 ± 6.4 g/L) and total protein (70.4 ± 6.9 g/L) than control group patients (p < 0.001). Most patients did not have any records of comorbidities (LBMI: 44.4% and control: 45.7%). The recorded associated comorbidities included chronic kidney disease (CKD), cerebrovascular accident (CVA), cerebrovascular disease (CVD), chronic obstructive pulmonary disease (COPD), diabetes (DM), liver diseases, connective tissue diseases, and IBD. More cases of control group patients had different types of diabetes: 121 (41.7%) compared to 28 (25.9%) patients in the LBMI group with a high odds ratio (OR = 2.05, 95% CI = 1.25-3.34, p = 0.004). These types included DM type 1 (5 [1.3%] and 4 [1%]), DM type 2 (22 [5.5%] and 117 [29.4%]), and diabetic ketoacidosis (DKA) (5 [1.3%] and 4 [1%]) in the LBMI group as well as DM type 2 (1 [0.3%] and 0 [0%]) in the control group, respectively. The results recorded one case of colorectal cancer (CRC), chronic myelogenous leukemia (CML), and pancreatic cancer in the LBMI group, whereas the control group had six cases of CRC and one case of hepatocellular carcinoma (HCC). The data on malignancy did not reveal any significant variation between both groups (p = 0.626). The percentage of IBD cases was significantly higher in the LBMI (15.7%) than in the control (5.2%) groups (OR = 0.29, 95% CI = 0.14-0.61, p = 0.001) (Table 1).

At admission, some patients (54%) had specific treatments, mainly for the comorbidities mentioned above. These treatments included chemotherapies, biological therapies, proton pump inhibitors (PPI), antibiotics, steroids, anticoagulants, or tube feeding. Biological therapies, steroids, and feeding tubes were significantly reported for the LBMI patients as 15.7%, 4.6%, and 16.7% compared to 5.2%, 0.7%, and 6.9% of the control (p < 0.05), respectively. None of the other treatments were significantly predominant in any of the tested groups (Table 1).

Before admission, different types of endoscopy were used in the studied patients, as stated in medical reports. The endoscopic interventions included esophagogastroduodenoscopy (EGD), endoscopic retrograde cholangiopancreatography (ERCP), percutaneous endoscopic gastrostomy (PEG), colonoscopy, sigmoidoscopy, or a combined procedure. Other reasons included mucosal breaching (biopsy/polypectomy during the procedure), band ligation, stent insertion, or therapy injection. In about 41.7% of LBMI and 37.2% of the control group patients' medical records, no clear intervention was mentioned. Except for PEG (OR = 0.175, 95% CI = 0.05-0.59, p = 0.004), none of the above-mentioned procedures had a significant majority in both groups. About 7.4% of the LBMI underwent PEG compared to 1.7% of the control group (Table 2).

**Table 2:** Endoscopic Intervention data according to the BMI category of the studied patients ^#^ One-Way ANOVA (POSTHOC = LSD ALPHA [0.05]). * Significant at p < 0.05. BMI: Body mass index; EGD: Esophagogastroduodenoscopy; ERCP: Endoscopic retrograde cholangiopancreatography; PEG: Percutaneous endoscopic gastrostomy; LSD: Least significant difference.

Variables	LBMI (N = 108)	Control (N = 290)	P-value^#^	OR	95% CI	
					Lower	Upper
No intervention	45 (41.7%)	108 (37.2%)	0.244	0.83	0.53	1.3
EGD	51 (47.2%)	133 (45.9%)	0.448	0.95	0.61	1.47
Colonoscopy	41 (38%)	122 (42.1%)	0.266	1.19	0.75	1.87
ERCP	3 (2.8%)	15 (5.2%)	0.232	1.91	0.54	6.73
PEG	8 (7.4%)	4 (1.4%)	0.004*	0.175	0.05	0.59
Combined procedure	3 (2.8%)	15 (5.2%)	0.232	1.91	0.54	6.73
Sigmoidoscopy	2 (1.9%)	1 (0.3%)	0.18	0.18	0.02	2.04
Polypectomy	7 (6.5%)	19 (6.6%)	0.592	1.01	0.41	2.48
Biopsy	53 (49.1%)	147 (50.7%)	0.431	1.07	0.69	1.66
Brand ligation	2 (1.9%)	5 (1.7%)	0.609	0.93	0.18	4.87
Stent insertion	3 (2.8%)	4 (1.4%)	0.288	0.49	0.11	2.22
Injection therapy	0 (0%)	0 (0%)	-	-	-	-

**Figure 1:**
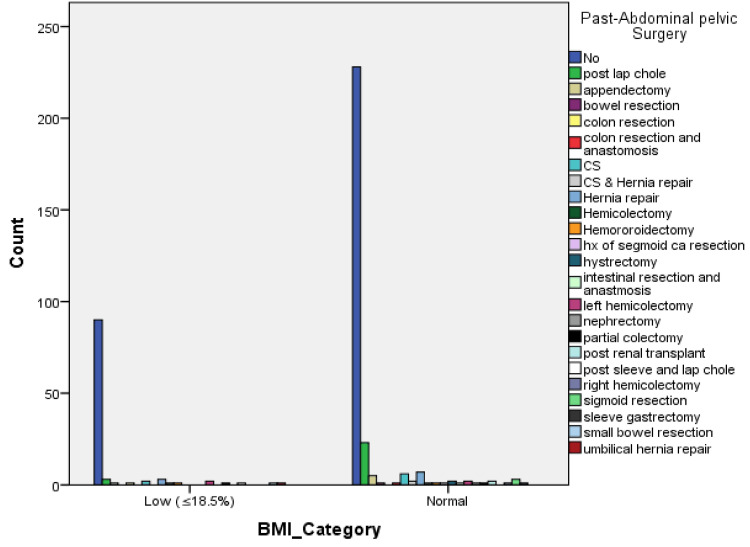
Bar charts of past-abdominal pelvic surgery in the studied patients

Several types and indications were reported in the history of past-abdominal pelvic surgeries in some patients (20.1%) (Figure 1). These included post-lap chole, post-sleeve, appendectomy, bowel resection, colon resection, anastomosis, C-section, hernia repair, hemicolectomy, hemorrhoidectomy, hysterectomy, intestinal resection, anastomosis, left hemicolectomy, right hemicolectomy, nephrectomy, colectomy, post renal transplant, sleeve gastrectomy, and sigmoid resection. There was insignificant variation between the two BMI groups (p = 0.452).

**Figure 2:**
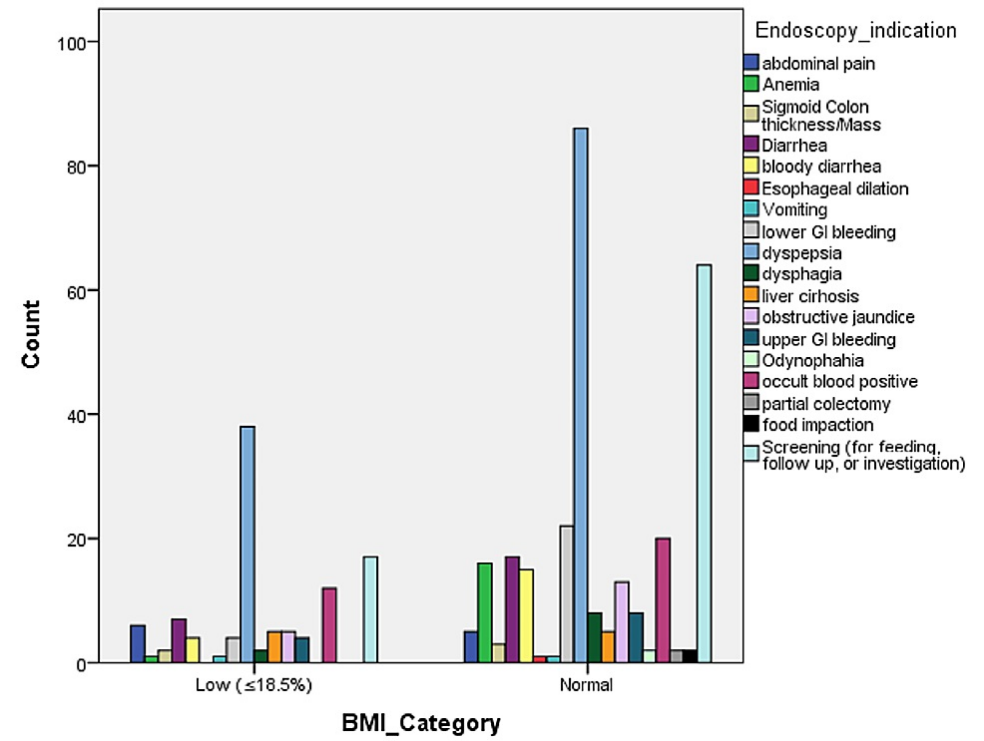
Bar charts of endoscopy indications in the studied groups

After admission, all patients underwent endoscopy procedures to investigate and diagnose different complications. The procedure was completed in 383 (96%) of patients, whereas only 15 (4%) had some complications because of poor preparation (OR = 0.66, 95% CI = 0.18-2.39). As shown in Table 3, endoscopy was indicated because of abdominal pain, anemia, sigmoid colon thickness/mass, diarrhea, bloody diarrhea, esophageal dilation, vomiting, lower or upper GI bleeding, dyspepsia, dysphagia, liver cirrhosis, obstructive jaundice, odynophagia, occult blood positive, a partial colectomy, food impaction, or just screening for feeding, follow-up, or investigation. There was no significant variation in the endoscopy indication between the two BMI groups (Figure 2).

**Table 3:** Endoscopic indication data according to the BMI category of the studied patients ^#^ One-Way ANOVA (POSTHOC = LSD ALPHA [0.05]). BMI: Body mass index; GI: Gastrointestinal; LSD: Least significant difference.

Variables	LBMI (N = 108)	Control (N = 290)	P-value^#^
Abdominal pain	6 (5.6%)	5 (1.7%)	0.225
Anemia	1 (0.9%)	16 (5.5%)	
Sigmoid colon thickness/Mass	2 (1.9%)	3 (1%)	
Diarrhea	7 (6.5%)	17 (5.9%)	
Bloody diarrhea	4 (3.7%)	15 (5.2%)	
Esophageal dilation	0 (0%)	1 (0.3%)	
Vomiting	1 (0.9%)	1 (0.3%)	
Lower GI bleeding	4 (3.7%)	22 (7.6%)	
Dyspepsia	38 (35.2%)	86 (29.7%)	
Dysphagia	2 (1.9%)	8 (2.8%)	
Liver cirrhosis	5 (4.6%)	5 (1.7%)	
Obstructive jaundice	5 (4.6%)	13 (4.5%)	
Upper GI bleeding	4 (3.7%)	8 (2.8%)	
Odynophagia	0 (0%)	2 (0.7%)	
Occult blood positive	12 (11.1%)	20 (6.9%)	
Partial colectomy	0 (0%)	2 (0.7%)	
Food impaction	0 (0%)	2 (0.7%)	
Screening (for feeding, follow-up, or investigation)	17 (15.7%)	64 (22.1%)	

Regarding endoscopy diagnosis, more accurate outcomes were denoted for patients of the two groups. Endoscopy findings were normal only in 16 (14.8%) and 49 (16.9%) of the LBMI and control groups, respectively, which indicated some asymptomatic disorders or previous misdiagnosis. The most common diagnostic findings were gastritis and internal hemorrhoid in 8.3% of the LBMI patients, followed by colonic polyps, Crohn’s disease, and esophageal varices (5.6%). In the control group patients, the most common findings were internal hemorrhoid (6.9%), colonic polyp (5.5%), GERD (5.5%), *H. pylori* (4.8%), gastritis (4.5%), Common bile duct (CBD) stones (4.5%), and nodular gastritis (3.4%) (Appendix table).

No post-endoscopy complications were reported in both groups. The screened complications were GI bleeding, aspiration pneumonia, systemic infection, GI perforation, or over-sedation. A comparison of these reports and outcomes confirmed a robust endoscopy technique used at the studied center, which avoided the most known risk factors for LBMI patients.

## Discussion

The present study sheds light on the relationship between LBMI and clinical outcomes after abdominal endoscopic operations. A review of medical records from 398 individuals having endoscopy revealed numerous noteworthy findings involving demographic features, clinical profiles, procedural details, and post-endoscopy outcomes.

One of the most notable results was that LBMI patients were much younger than those in the control group. This study implies that younger people may be more vulnerable to variables that contribute to LBMI, such as chronic illnesses or dietary inadequacies, which could affect their risk profile for endoscopic treatments. A previous study showed that females with a BMI < 19 or males with a BMI < 20 were at high risk of primary vascular regulation, Flammer syndrome, systemic hypoxia, eye disorders, depression, multiple sclerosis, xerostomia, chronic inflammations, and a predisposition to cancer; however, those were not recorded in the current study [18]. In a cross‐sectional study conducted by Katanoda et al., they found that female adults at younger ages of 18 years with low BMI were at higher risk of diabetes with a significantly high odds ratio of 2.25 and 95% CI: 1.01-4.99 [19].

Furthermore, the current study found disparities in laboratory markers between the two BMI groups, with lower amounts of albumin and total protein in individuals with LBMI. Furthermore, LBMI patients suffered from CVA and connective tissue diseases more than patients with a higher BMI. These biochemical markers reflect nutritional status and overall health, emphasizing the susceptibility of LBMI patients to malnutrition-related problems. In the study conducted by Streng et al., LBMI patients had higher natriuretic peptide levels and lower levels of uric acid, pro-adrenomedullin, creatinine, sodium, and bicarbonate [20].

In the current study, comorbidities were distributed differently among the groups, with diabetes mellitus being more common in the control group. Interestingly, IBD was more common among LBMI patients, implying a link between BMI and GI pathology. Several studies highlighted the relationship between LBMI and IBD factors, such as morbidity, complications, prognosis, disease stages, and available therapy. It was reported before that IBD patients usually had an LBMI [21], which increases the risk of Crohn’s disease [22,23]. The unequal distribution of specific comorbidities emphasizes the significance of taking underlying medical problems into account when calculating the likelihood of adverse outcomes following endoscopic treatments.

Treatment techniques vary between BMI groups, with LBMI patients using more biological treatments, steroids, and feeding tubes. These therapies may represent the treatment of underlying diseases such as IBD or malnutrition, which might affect procedure results. Furthermore, endoscopic interventions vary per patient, with the most prevalent procedures being esophagogastroduodenoscopy (EGD) and colonoscopy. Importantly, no significant variations in the distribution of endoscopic indications were seen across the BMI groups, suggesting that the choice to conduct endoscopy was unaffected by BMI status. Endoscopy detected a variety of GI diseases, including gastritis, colonic polyps, Crohn's disease, and esophageal varices. Importantly, no post-endoscopy problems were observed in either BMI group, demonstrating that the research center's endoscopic procedures are generally safe.

Against our findings, previous studies demonstrated that LBMI patients, who underwent EGD, PEG, and colonoscopy, were at high risk of endoscopy-related SAEs, such as GI bleeding, perforation, and aspiration pneumonia [15,24]. Another study from Milton Keynes University Hospital, UK, showed that LBMI patients had a high incomplete colonoscopy rate, particularly for the sigmoid colon, which might be because of patient intolerance and old age (>40 years) [25]. Similar to our findings, a cohort study from California, USA, showed that LBMI did not increase the incidence or severity of post-ERCP pancreatitis, a common complication after an endoscopic procedure [26]. These discrepancies may indicate variations in medical center facilities and the efficiency of medical teams in managing post-endoscopic complications.

The study was based on retrospective data collection. One of the most significant drawbacks of the retrospective was misclassification/ascertainment bias. Retrospective studies rely on data that were recorded into a clinical database but not collected for study. The investigator had little control over the type and quality of the gathered variables, and they could not guarantee that the features of interest were correctly and reliably documented. Furthermore, certain variables that may influence the conclusion may not have been captured at all. The patient records are likely to be incomplete in various ways and may contain incorrect data.

## Conclusions

The link between low BMI and post-endoscopy SAEs emphasizes the significance of individualized risk assessment and mitigation techniques in this vulnerable patient population. The current study revealed that abdominal pain, diarrhea, dyspepsia, and positive occult blood are the most common indications for LBMI patient's endoscopy. Also, colonic polyps, Crohn’s disease, esophageal varices, gastritis, and internal hemorrhoids were the most common diagnostic pathologies for LBMI patients. Pre-procedural optimization, such as nutritional support and hydration, may enhance results in those with a low BMI, who are undergoing endoscopy. Furthermore, careful choice of endoscopic procedures and equipment as well as close monitoring during and after the surgery are required to reduce the chance of adverse effects. The current study was limited to a small sample size from a single medical center. So, future research should concentrate on prospective studies with larger sample sizes or multiple medical centers to corroborate these findings and identify strategies to lower post-endoscopy problems' prevalence in patients with low BMI. Overall, the findings of this study help us comprehend the complicated interplay between BMI, clinical features, and outcomes after abdominal endoscopy. While low BMI may be linked with specific risk factors and comorbidities, thorough pre-procedural evaluation and adequate patient care can significantly reduce the risk of AE and provide the best possible operative results.

## Appendices

**Table 4:** Endoscopic diagnosis outcomes according to the BMI category of the studied patients ^#^ One-Way ANOVA (POSTHOC = LSD ALPHA [0.05]). BMI: Body mass index; GI: Gastrointestinal; CBD; Common bile duct; GERD: Gastroesophageal reflux disease; LSD: Least significant difference.

Variables	LBMI (N = 108)	Control (N = 290)	P-value^#^
Not done	2 (1.9%)	8 (2.8%)	0.715
Normal	16 (14.8%)	49 (16.9%)	
Nodular gastritis	2 (1.9%)	10 (3.4%)	
Nodular gastritis and hiatal hernia	0 (0%)	1 (0.3%)	
Antral gastritis and erosive duodenitis	1 (0.9%)	0 (0%)	
Barrett's esophagus	0 (0%)	2 (0.7%)	
CBD stone extraction and stent insertion	5 (4.6%)	13 (4.5%)	
Celiac disease	5 (4.6%)	8 (2.8%)	
Colitis	(0%)	10 (3.4%)	
Colorectal cancer	1 (0.9%)	2 (0.7%)	
Colonic polyp	6 (5.6%)	16 (5.5%)	
Colonic polyp and internal hemorrhoid	1 (0.9%)	1 (0.3%)	
Colonic polyp and sigmoid mass	0 (0%)	1 (0.3%)	
Diverticulosis	0 (0%)	7 (2.4%)	
Crohn’s disease	6 (5.6%)	5 (1.7%)	
Diffuse antral erythema/gastritis	1 (0.9%)	1 (0.3%)	
Duodenal ulcer	1 (0.9%)	4 (1.4%)	
Post-renal transplant	1 (0.9%)	1 (0.3%)	
Eosinophilic esophagitis	1 (0.9%)	0 (0%)	
Esophageal cancer	0 (0%)	1 (0.3%)	
Esophageal candida	0 (0%)	2 (0.7%)	
Esophageal varices	6 (5.6%)	7 (2.4%)	
Esophageal polyps	1 (0.9%)	0 (0%)	
Esophageal food impaction pushed to the stomach	0 (0%)	1 (0.3%)	
Esophageal ring and gastritis	0 (0%)	1 (0.3%)	
Esophageal stricture and esophagitis	0 (0%)	1 (0.3%)	
GERD	5 (4.6%)	16 (5.5%)	
GERD and gastritis	0 (0%)	3 (1%)	
GERD and internal hemorrhoid	0 (0%)	2 (0.7%)	
GERD and erosive duodenitis	0 (0%)	1 (0.3%)	
Erosive gastritis	2 (1.9%)	4 (1.4%)	
Erosive gastritis and hiatal hernia	1 (0.9%)	1 (0.3%)	
Gastritis	9 (8.3%)	13 (4.5%)	
Gastritis and colonic polyp/mass	2 (1.9%)	2 (0.7%)	
Gastritis and *H. pylori*	4 (3.7%)	14 (4.8%)	
Gastro-duodenitis	3 (2.8%)	5 (1.7%)	
Gastro-duodenitis and hiatal hernia	0 (0.0%)	1 (0.3%)	
Gastritis and hiatal hernia	0 (0.0%)	2 (0.7%)	
Gastritis and angiodysplasia	1 (0.9%)	0 (0%)	
Gastritis and esophageal candida	0 (0%)	1 (0.3%)	
Gastritis, esophagitis, and internal hemorrhoid	0 (0%)	1 (0.3%)	
Gastritis and internal hemorrhoid	0 (0%)	1 (0.3%)	
Hiatal hernia	2 (1.9%)	4 (1.4%)	
Gastric ulcer	1 (0.9%)	2 (0.7%)	
Internal hemorrhoid	9 (8.3%)	20 (6.9%)	
External hemorrhoid	0 (0%)	3 (1%)	
Remission of ulcerative colitis	1 (0.9%)	4 (1.4%)	
Relapse of ulcerative colitis	1 (0.9%)	7 (2.4%)	
Relapse Crohn’s disease	0 (0%)	2 (0.7%)	
Rectosigmoid mass (malignant/mostly malignant)	0 (0%)	8 (2.8%)	
Ulcerative colitis	3 (2.8%)	4 (1.4%)	
Sessile polyp, and internal hemorrhoid	0 (0%)	1 (0.3%)	
Variceal bleeding	1 (0.9%)	3 (1%)	
Variceal bleeding and hiatal hernia	1 (0.9%)	1 (0.3%)	
Reflex esophagitis and hiatal hernia	0 (0%)	1 (0.3%)	
Plummer-Vinson syndrome	0 (0%)	1 (0.3%)	
Scattered erythema of sigmoid colon	0 (0%)	1 (0.3%)	
Large bleeding internal piles	0 (0%)	1 (0.3%)	
Others	6 (5.6%)	8 (2.7%)	
